# Maternal and Fetal Outcomes among Pregnant Women with Diabetes

**DOI:** 10.3390/ijerph19063684

**Published:** 2022-03-20

**Authors:** Miroslava Gojnic, Jovana Todorovic, Dejana Stanisavljevic, Aleksandra Jotic, Ljiljana Lukic, Tanja Milicic, Nebojsa Lalic, Katarina Lalic, Milica Stoiljkovic, Tamara Stanisavljevic, Aleksandar Stefanovic, Katarina Stefanovic, Svetlana Vrzic-Petronijevic, Milos Petronijevic, Zorica Terzic-Supic, Maja Macura, Milan Perovic, Sandra Babic, Pavle Piperac, Marija Jovanovic, Bijana Parapid, Krisitna Doklestic, Radmila Cerovic, Sinisa Djurasevic, Stefan Dugalic

**Affiliations:** 1 Clinic for Obstetrics and Gynecology, University Clinical Centre of Serbia, 11000 Belgrade, Serbia; miroslavagojnicdugalic@yahoo.com (M.G.); aleksandar.stefanovic@med.bg.ac.rs (A.S.); jeremick@hotmail.com (K.S.); vrzic.dr@gmail.com (S.V.-P.); ordinacija.petronijevic@gmail.com (M.P.); maja_macura@live.com (M.M.); sandradrmilic@yahoo.com (S.B.); radojka.cerovic@gmail.com (R.C.); 2 Faculty of Medicine, University of Belgrade, 11000 Belgrade, Serbia; aleksandra.z.jotic@gmail.com (A.J.); ljlukic@eunet.rs (L.L.); icataca@gmail.com (T.M.); nebojsa.lalic@med.bg.ac.rs (N.L.); katarina.lalic@med.bg.ac.rs (K.L.); mmstoiljkovic@yahoo.com (M.S.); stamara8@yahoo.com (T.S.); 3 Institute of Social Medicine, Faculty of Medicine, University of Belgrade, 11000 Belgrade, Serbia; jovana.todorovic@med.bg.ac.rs (J.T.); zorica.terzic-supic@med.bg.ac.rs (Z.T.-S.); 4 Institute for Medical Statistics and Informatics, Faculty of Medicine, University of Belgrade, 11000 Belgrade, Serbia; dejana.stanisavljevic@med.bg.ac.rs; 5 Clinic for Endocrinology, Diabetes and Metabolic Diseases, University Clinical Centre of Serbia, 11000 Belgrade, Serbia; 6 Clinic for Gynecology and Obstetrics “NarodniFront”, Faculty of Medicine, University of Belgrade, 11000 Belgrade, Serbia; perovicmilan@hotmail.com; 7 Department for Humanities, Faculty of Medicine, University of Belgrade, 11000 belgrade, Serbia; pavle.piperac@med.bg.ac.rs; 8 General Hospital Bor, 19210 Bor, Serbia; marija.jovanovic50.10@gmail.com; 9 Clinic for Cardiology, Faculty of Medicine, University of Belgrade, 11000 Belgrade, Serbia; bilja-na_parapid@yahoo.com; 10 Emergency Department, University Clinical Centre of Serbia, 11000 Belgrade, Serbia; kristinadoklestic@gmail.com; 11 Faculty of Biology, University of Belgrade, 11000 Belgrade, Serbia; sine@bio.bg.ac.rs

**Keywords:** diabetesin pregnancy, pre-gestational diabetes, gestational diabetes

## Abstract

The aim of this study was to examine the differences in pregnancy complications, delivery characteristics, and neonatal outcomes between women with type 1 diabetes mellitus (T1DM), type 2 diabetes mellitus (T2DM), and gestational diabetes mellitus (GDM). This study included all pregnant women with diabetes in pregnancy in Belgrade, Serbia, between 2010 and 2020. The total sample consisted of 6737 patients. In total, 1318 (19.6%) patients had T1DM, 138 (2.0%) had T2DM, and 5281 patients (78.4%) had GDM. Multivariate logistic regression with the type of diabetes as an outcome variable showed that patients with T1DM had a lower likelihood of vaginal delivery (OR: 0.73, 95% CI: 0.64–0.83), gestational hypertension (OR: 0.47, 95% CI: 0.36–0.62), higher likelihood of chronic hypertension (OR: 1.88, 95% CI: 1.55–2.29),and a higher likelihood ofgestational age at delivery before 37 weeks (OR: 1.38, 95% CI: 1.18–1.63) compared to women with GDM. Multivariate logistic regression showed that patients with T2DM had a lower likelihood ofgestational hypertension compared to women with GDM (OR: 0.37, 95% CI: 0.15–0.92).Our results indicate that the highest percentage of diabetes in pregnancy is GDM, and the existence of differences in pregnancy complications, childbirth characteristics, and neonatal outcomes are predominantly between women with GDM and women with T1DM.

## 1. Introduction

Diabetes mellitus (DM) represents an enormous public health problem worldwide. According to the International Diabetes Federation (IDF), around 10% of global health expenditure is spent on diabetes, and by the year 2045, the number of people diagnosed with diabetes will rise to 700 million [1]. It is estimated that by 2030, the age-adjusted prevalence of DM in adults willbe 9.2% worldwide and 7.3% in Europe compared to 8.3% and 6.3%, respectively, in 2019. In addition, DM raises a lot of global health care inequity concerns. In Balkan countries, North Macedonia has the highest prevalence of DM (around 9.3%), followed by Serbia, Albania, Montenegro, and Bosnia and Herzegovina, with 9.0% of the population affected with diabetes, while Romania hasa rate of 6.9%, followed by Croatia (6.4%), Bulgaria (6.0%), and Slovenia (5.9%),andGreece has the lowest prevalence—around 4.7% [2].

There is a concerning increase in the prevalence of diabetes during pregnancy, along with anincrease in the prevalence of diabetes in the general population [3]. Around 1 in 6 live births are affected by hyperglycemia in pregnancy, and almost 85% of these patients suffer from gestational diabetes mellitus (GDM), with the remaining being pre-gestational diabetes [4,5,6]. GDM is defined as:“glucose intolerance with onset or first recognition during pregnancy” [7]. The prevalence of GDM varies between countries, ranging from 17.3 to 25.5% in the USA [7], down to 13.2% in Germany [8], and 11.5% in pooled prevalence among Asian countries [9]. To the best of our knowledge, there are no data about the incidence or prevalence of GDM and pre-gestational diabetes in Balkan countries, including the Republic of Serbia.

There is a different age distribution among pregnant womenwith DM type 1 (T1DM), DM type 2 (T2DM), and GDM. Women withT2DM and GDM are usually older thanwomen with T1DM [10,11]. This can be explained bythepathophysiology of disease development for each DM type.

Due to the insulin resistance in pregnancy, beta cell hypertrophy occurs, but when insulin secretion cannot match the increased insulin demands due to the pregnancy-induced insulin resistance, gestational diabetes occurs. The glucose is then transferred through the placenta and leads to concomitant fetal hyperglycemia, followed by the hyperplasia of the beta-cells in the fetal pancreas and fetal hyperinsulinemia. This leads to excessive weight gain (≥4000 g) in the fetus, as a consequence of fetal hyperinsulinemia, usually known as large-for-gestational-age (LGA) infants or fetal macrosomia [12,13]. Women with pre-gestational diabetes involves both T1D and T2D and may differ in their ability to produce insulin. Some can probably increase their insulin secretion, but still not enough to compensate for the increase in insulin resistance [14]. In well-controlled T1DM with normal glucose levels during the first trimester and throughout pregnancy, and small variations in blood glucose levels (first-trimester HbA1c levels  <8.5%), there is no higher risk for LGA infants compared to non-diabetic mothers [15,16]. On the other end of the spectrum, women with DM sometimes have pregnancies complicated by intrauterine growth restriction (IUGR). This complication of pregnancy has a more complex pathophysiological mechanism behind it. Essentially, DM is a risk factor for the development of hypertensive disorders of pregnancy. Due to abnormal spiral artery remodeling, placentation in these patients is poor, leading to a lack of blood flow to the fetus and subsequent IUGR [17].

Different factors predispose the development of hypertensive disorders during pregnancy in mothers with DM. First of all, younger women and those suffering from T1DM usually do not have chronic hypertension, unlike older women and those suffering from T2DM. However, in arecent study published by Morikawa et al. [18], there was no statistical difference between these two groups in the rates of hypertensive disorders in pregnancy (HDP), including preeclampsia.

The pathophysiological mechanisms of HDP in diabetic mothers are still a matter of debate. Even though all pregnant women have some degree of insulin resistance (IR), among women with diabetes, maternal adaptation in insulin sensitivity increases oxidative stress levels. The common denominator for both DM and HDP are inflammation and oxidative stress that lead to endothelial dysfunction and poor placentation in the case of HDP, including gestational hypertension, preeclampsia, and HELLP syndrome [19,20]. In addition, lower placental volume at 11–14 GW is considered an independent predictor of preeclampsia and IUGR [21]. Withthis in mind, DM with poor control in the first trimester can lead to HDP and subsequent IUGR independently of DM type, while pregnanciescomplicated only by DM without superimposed HDP usually lead to the development of fetal macrosomia. Preterm delivery and operative delivery are more common among women with diabetes in pregnancy [11,22].

Along with the macrosomia, IUGR, and HDP in pregnancy, diabetes in pregnancy is associated with spontaneous abortions, a higher likelihood for Cesarean deliveries andoperative vaginal deliveries, but also higher general perinatal mortality, including perinatal asphyxia and congenital anomalies, especially involving cardiac anomalies, polycythemia, organomegaly, neonatal hypoglycemia, changes in electrolytes, and neonatal hyperbilirubinemia [23,24,25,26,27].

There is a lack of studies on the differences in co-morbidities and the differences in both maternal pregnancy outcomes and fetal pregnancy outcomes between women with different types of diabetes in pregnancy. The aim of this study was to examine the differences in pregnancy complications, delivery characteristics, and neonatal outcomes between women with T1DM, T2DM, and GDM.

## 2. Materials and Methods

This study used data from the Birth database for Belgrade from the City Institute of Public Health. The study included all pregnant women with diabetes in pregnancy in Belgrade, Serbia, treated in any health care institution between 2010 and 2020. The total sample consisted of 6737 patients.

The database provides data for the socio-demographic data (maternal age in years) and clinical data on the pregnancy characteristics (presence of any complication in pregnancy, e.g., HEELP syndrome, gestational hypertension, chronic hypertension, preeclampsia), delivery characteristics (type of delivery, gestational age at delivery in weeks), and newborns’ characteristics (birth weight in grams, birth height in centimeters, Apgar score).

Based on the type of diabetes, women were classified into three groups: women with T1DM-1318 (19.6%), women with T2DM-138 (2.0%), and women with GDM-5281 (78.4%).

According to the guidelines for diagnosing and treating diabetes in Serbia, all women without a prior diagnosis of diabetes are referred to the Oral Glucose Tolerance Test between the 24th and 28th week of gestation, with 75 g of glucose. The diagnosis of GDM is established based on the IADPSG criteria according to these guidelines as of 2012. Before the guidelines, which were issued in 2012 Serbia, the guidelines used were from 2002, and the diagnosis of GDM was established with 100/g OGTT with the cutoffs of 5.5 mmol/L for fasting glucose and 9 mmol/L for two hours after OGTT glucose level [28,29]. Based on the age in years, women were classified into two groups: age of 34 and under and age over 34, as the maternal age of above 34 is considered a risk factor for diabetes in pregnancy [20]. Based on the newborns’ birth weight and gestational age at delivery (in weeks), newborns were classified as small for gestational age newborns (SGA), appropriate for gestational age newborns (AGA),orlarge for gestational age (LGA). We used the percentiles to classify the infants in these categories. The infants whose birth weight was below the 10th percentile for the gestational age at delivery were classified as SGA, the infants whose birth weight was between the 10th and 90th percentile for thegestational age at delivery were classified as AGA, and thoseabove the 90th percentile were classified as LGA, based on the available percentiles of the World Health Organization (WHO) [30,31]. Ponderal index (PI=) was calculated as [32]:

PI= birth weight × 100/(birth height in centimeters)^3^.

The Ethical Committee of the Faculty of Medicine, University of Belgrade, approved this study (No. 1322/IX-80).

Statistical analyses were completedusing descriptive and analytical statistics. The differences between the groups were examined using the Chi-square test for qualitative variables and one-way ANOVA for numerical variables. All variables thatwere shown to be significant were entered into multivariate logistic regression analyses, with the type of diabetes as an outcome variable and GDM as a reference category. All statistical analyses were completedin the Statistical Software for Social Sciences SPSS 22.0.

## 3. Results

A total of 6737 womenwith diabetes in pregnancy was included in the study. Among them, 1318 (19.6%) had T1DM, 138 (2.0%) had T2DM, and 5281 women (78.4%) had GDM. Women with T1DM, T2DM, and GDM differed significantly in the average age (32.79 ± 5.41 vs. 31.88 ± 5.38 vs. 33.02 ± 5.17, *p* = 0.018); in the frequency of vaginal delivery; gestational hypertension; chronic hypertension;status of an Apgar score under 8; frequency of SGA, AGA, and LGA; and frequency ofgestational age before 37 weeks at delivery. The characteristics of womenfrom all three groups are presented in Table 1.

Multivariate logistic regression analyses with the type of diabetes as an outcome variable showed that women with T1DM had a lower likelihood of vaginal delivery (OR: 0.73, 95% CI: 0.64–0.83)andgestational hypertension (OR: 0.47, 95% CI: 0.36–0.62), and a higher likelihood of chronic hypertension (OR: 1.88, 95% CI: 1.55–2.29) and gestational age at delivery before37 weeks (OR: 1.38, 95% CI: 1.18–1.63) compared to women with GDM.

Multivariate logistic regression analyses with the type of diabetes as an outcome variable showed that women with T2DM had a lower likelihood ofgestationalhypertension compared to the women with GDM (OR: 0.37, 95% CI: 0.15–0.92). The results of the multivariate analyses with the type of diabetes as an outcome variableare presented in Table 2.

## 4. Discussion

We studied the differences in pregnancy complications, delivery characteristics, and neonatal outcomes between women with T1DM, women with T2DM, and women with GDM using data from the registry for all patients in Belgrade for 11 consecutive years and a sample of almost 7000 pregnant women. This study is based on the registry data, while the majority of studies examining diabetes in pregnancy are based in the clinical settings, which can be afactor influencing the prevalence and the characteristics of the population in the studies.As in the previous studies, the majority of womenwith diabetes in our study had GDM, around four-fifths of women, in accordance with the reports from other countries and large centers in which GDM comprisedbetween 80% and 90% of all cases of diabetes in pregnancy [4,5,33]. Our study included the registry data that contained data on all deliveries in Belgrade, like the study of Khalifeh et al.,whichincluded the data for the city of Dublin, Ireland. The study in Dublin showed aprevalence of pre-gestational diabetes of 20% and 18% in two time periods (1999–2003 and 2004–2008, respectively) among pregnant women with diabetes [34], almost identical to our results. Another regional study from Tuscany, Italy, showed arelatively high attribution of GDM to DM in pregnancy of 95.7% [35]. The study that included the data on more than 1.5 million live births in Sweden between 1998 and 2012 showed that the percentage of GDM among diabetes in pregnancy was just under 75% and that the prevalence of T2DM was 16.6% [36]. The data from the USA showed a similar prevalence of GDM among diabetes in pregnancy, but also ahigh prevalence of T2DM in pregnancy of more than 16%, with less than 2% of diabetes in pregnancy being T1DM [37]. Only 2% of the patients in our study had T2DM. However, as there is a concerning trend worldwide ofanincreaseinthe maternal age and prevalence of maternal obesity, there is a justified concern that the prevalence of T2DM in pregnancy may increase in future decades [38]. The lower prevalence of T2DM among women with diabetes in our study may be due to the lower fertility rates of women with pre-gestational T2DM and its association with obesity and polycystic ovarian syndrome, both contributing to the fertility decrease [6]. On the other hand, a certain percentage of women with pre-gestational T2DM may have been classified as GDM, as many patients may have been screened for glucose intolerance for the first time during pregnancy, and the pre-gestational T2DM may have been missed [39]. The data are sent to the registry right after the delivery, and there are no data on the six-week post-partum OGTT that can help clarify the final diagnosis.The data on diabetes in pregnancy in the low and middle-income countries that Serbia belongs to aregenerally sparse. As shown in one systematic review, very few studies examined the prevalence of pre-gestational diabetes in these countries and the total attribution of pre-gestational diabetes to diabetes in pregnancy [40].

The results of our study indicate the differences between women with different types of diabetes in pregnancy and suggest the adjustment of clinical approaches to these patients. Women with T1DM in our study had a higher likelihood of having chronic hypertension than women with GDM and a higher likelihood of preterm birth, which is in accordance with the previous reports on the association between chronic hypertension and preterm birth [19]. As diabetes and hypertension are associated with oxidative stresses, there is a higher likelihood ofdeveloping hypertension with alonger duration of diabetes. On the other hand, women with T1DM had more than a two-timeslower likelihood of developing gestational hypertension than women with GDM. The lower likelihood ofgestational hypertension among women with T1DM may be explained as a bias due to the high frequency of pre-pregnancy chronic hypertension in this population, as its prevalence was almost double among women with T1DM compared to women with GDM. Similarly, women with T2DM had an almost three-times lower likelihood ofgestational hypertension compared to women with GDM, which may also be reflected in the higher frequency of chronic hypertension prior to pregnancy, rather than the reduced risk for cardiovascular complications being pregnancy-related. However, there could also be a possible association betweengestational hypertension and the late diagnosis of GDM. As per the current recommendations [28,29], the screening for GDM is conducted between the 24th and 28th weeks of gestation. A recent study in the Slavic population showed that pregnant women in the first trimester have insulin resistance similar or higher in level to the insulin resistance present in the non-pregnant women with polycystic ovarian syndrome. The insulin resistance and early development of GDM that is undiagnosed and untreated may also contribute to the development of gestationalhypertension, which may indicate the need for earlier screening for GDM in this population [41]. In any case, one in six women in our study had some form of hypertensive disorder in pregnancy, indicating the high importance of adequate blood pressure control among pregnant women with diabetes along with strict glucose control. Close monitoring and the timely introduction of dietary advice and treatment may lower this incidence and improve the overall pregnancy outcomes. The advice should be individualized to each patient and based on their personal health history [42].

Women with T1DM had a 38% higher likelihood of preterm birth compared to women with GDM, and the frequency of preterm birth was higher among women in this group compared to both T2DM and GDM, which is in accordance with the previous studies examining the differences in pregnancy outcomes among women with T1DM and women with T2DM [3]. There were no differences in the likelihood of different categories of newborns’ birth weight. However, as there was a higher likelihood of preterm delivery among women with T1DM and more than 25% lower likelihood of vaginal delivery in this group of women, this can reflect obstetric intervention and elective Cesarean delivery, not adecreased risk for macrosomia. Since we did not find the differences between the groups in the likelihood for adecreased Apgar score (Apgar score under 8), the timeliness of the obstetric intervention may be deemed appropriate in our study.

This study has a few possible limitations. The first is in its design, as the data were obtained from the patient history records, and we cannot establish a causal relationship between the variables. Additionally, the registry does not report the data on glycemic control, and we could not examine its relationship to both maternal and fetal outcomes. The registry is only city-based, and the results of our study cannot be generalized to the entire population of women with diabetes in pregnancy. The data are from the largest city in Serbia and the region, and many women from the entire region deliver their newborns in Belgrade and are therefore included in our data. This is the first study examining the maternal and fetal outcomes among women with diabetes in pregnancy in Serbia. We used the registry data for all patients treated in Belgrade for more than one decade, which is our biggest strength.

## 5. Conclusions

Our results indicate that the highest percentage of diabetes in pregnancy is GDM, as well as the existence of differences in pregnancy complications, childbirth characteristics, and neonatal outcomes, predominantly between women with GDM and women with T1DM. Compared to women with GDM, women with T1DM showeda higher likelihood of chronic hypertension and delivery at the gestational age before 37 weeks, with alower likelihood of vaginal delivery. Compared to women with GDM, women with pre-gestational diabetes had a lower likelihood of gestational hypertension. The effects of diabetes in pregnancy, chronic hypertension, and gestational hypertension on pregnancy outcomes, individually and, especially when combined, should be thoroughly examined, considering the increasing prevalence of diabetes in pregnancy.

## Figures and Tables

**Table 1:** Characteristics of womenin the three examined groups.

Characteristics	T1DM N (%)	T2DM N (%)	GDM N (%)	*p*-Value
**Age**				
<34 years	726 (55.1)	87 (63.0)	2827 (53.5)	
>34 years	592 (44.9)	51 (37.0)	2454 (46.5)	0.060
**Birth Weight**				
SGA	119 (9.0)	7 (5.1)	266 (5.0)	
AGA	905 (68.7)	103 (74.6)	3806 (72.1)	
LGA	294 (22.3)	28 (20.3)	1209 (22.9)	<0.001
**Delivery**				
Vaginal	565 (42.9)	70 (50.7)	2756 (52.2)	
Cesarean delivery	753 (57.1)	68 (49.3)	2525 (47.8)	<0.001
**Chronic Hypertension**				
No	1141 (86.6)	124 (89.9)	4918 (93.1)	
Yes	177 (13.4)	14 (10.1)	363 (6.9)	<0.001
**Preeclampsia**				
No	1291 (98.0)	137 (99.3)	5208 (98.6)	
Yes	27 (2.0)	1 (0.7)	73 (1.4)	0.154
**HELLP**				
No	1318 (100.0)	138 (100.0)	5279 (100.0)	
Yes	0 (0)	0 (0)	2 (0)	0.759
**Gestational Hypertension**				
No	1260 (95.6)	133 (96.4)	4789 (90.7)	
Yes	58 (4.4)	5 (3.6)	492 (9.3)	<0.001
Ponderal index (X ± SD)	2.55 ± 0.26	2.55 ± 0.27	2.50 ± 0.24	<0.001
**Apgar Score**				
<8	313 (24.0)	28 (20.3)	960 (18.3)	
>8	992 (76.0)	110 (79.7)	4297 (81.7)	<0.001
**Birth Length (in cms)**	50.84 ± 3.56	51.11 ± 2.95	51.65 ± 3.00	<0.001
**Gestational Age at Birth**	38.05 ± 2.24	38.53 ± 1.78	38.45 ± 1.75	<0.001
<37 weeks	325 (24.7)	19 (13.8)	912 (17.3)	
>37 weeks	993 (75.3)	119 (86.2)	4369 (82.7)	<0.001

SGA—small for gestational age; AGA—adequate for gestational age; LGA—large for gestational age; HELLP—hemolysis, elevated liver enzymes, and low platelets syndrome.

**Table 2:** Multivariate logistic regression analysis with the type of diabetes as an outcome variable and GDM as a reference category.

Characteristics	T1DM * OR (95% CI)	T2DM * OR (95% CI)
**Weight**		
SGA	1.29 (0.96–1.73)	1.38 (0.54–3.49)
AGA	0.94 (0.81–1.10)	1.20 (0.78–1.83)
LGA	1.0 reference category	1.0 reference category
**Delivery**		
Vaginal	**0.73 (0.64–0.83)**	1.00 (0.71–1.43)
Cesarean delivery	1.0 reference category	1.0 reference category
**Chronic Hypertension**		
No	1.0 reference category	1.0 reference category
Yes	**1.88 (1.55–2.29)**	1.48 (0.84–2.60)
**Gestational Hypertension**		
No	1.0 reference category	1.0 reference category
Yes	**0.47 (0.36–0.62)**	**0.37 (0.15–0.92)**
**Apgar Score**		
<8	1.13 (0.96–1.33)	1.20 (0.76–1.89)
>8	1.0 reference category	1.0 reference category
**Gestational Age at Birth**		
<37 weeks	**1.38 (1.18–1.63)**	0.70 (0.41–1.20)
>37 weeks	1.0 reference category	1.0 reference category

* Compared to the patients with gestational diabetes.SGA—small for gestational age; AGA—adequate for gestational age; LGA—large for gestational age.

## Data Availability

Not applicable.
